# Contributing factors to quality of life after vertebral artery dissection: a prospective comparative study

**DOI:** 10.1186/s12883-019-1541-x

**Published:** 2019-12-04

**Authors:** Rainer J. Strege, Reinhard Kiefer, Manfred Herrmann

**Affiliations:** 1 Department of Neurology, AGAPLESION Diakonieklinikum Rotenburg, Elise-Averdieck-Str.17, 27356, Rotenburg, Germany; 2 Center for Cognitive Sciences, Department of Neuropsychology and Behavioral Neurobiology, University of Bremen, Hochschulring 18, D-28359 Bremen, Germany

**Keywords:** Vertebral artery dissection, Acute ischemic stroke, Quality of life, Functional outcome, Cognitive symptoms, Posttraumatic stress symptoms

## Abstract

**Background:**

Vertebral artery dissection (VAD) may cause cerebral ischemia and impair quality of life (QOL) despite of good functional outcome. The aim of this study was the multimodal analysis of patient characteristics after VAD to identify contributing factors.

**Methods:**

In an exploratory study, 34 consecutive patients with first-ever spontaneous VAD were prospectively examined in comparison to 38 patients with cerebral ischemia without dissection and 25 stroke mimics as control groups. Multimodal assessment was performed for clinical, neurological, cognitive, psychological and radiological data at baseline and for QOL, functional outcome, and stress symptoms by questionnaire at six months follow-up. Subgroup analysis stratified for QOL by Stroke Specific Quality of Life Scale (SS-QOL) were done for patients with good functional outcome (modified Ranking Scale (mRS) scoring 0–2). Predictors for QOL at follow-up were analyzed by regression model.

**Results:**

88.2% of patients with VAD suffered from acute cerebral ischemia. Thirteen of 32 VAD patients (40.6%) rated QOL at follow-up as bad (SS-QOL score ≤ 3.9) despite of good functional outcome (mRS score 0–2). Subgroup analysis yielded significantly higher scores for posttraumatic stress symptoms (*p* = 0.002) in this subgroup. Posttraumatic stress symptoms, severity of neurological disorders, and impaired neuropsychological baseline performance proved to be independent predictors for reduced QOL at follow-up according to regression analysis.

**Conclusion:**

VAD leads to impaired QOL at 6 months follow-up due to multiple factors. The data suggest that posttraumatic stress symptoms are of significant importance for the QOL after VAD. Clinical monitoring should address this topic to make timely treatment possible.

## Background

Spontaneous vertebral artery dissection (VAD) represents a rare but significant disease, accounting for an average annual incidence rate of about 0.97 to 1.5 cases per 100.000 population [1, 2]. To diagnose VAD may be difficult because of the wide range of symptoms, from isolated local signs to posterior circulation stroke. Depending from the availability of imaging techniques such as magnetic resonance imaging (MRI) angiography as well as the awareness of physicians VAD has been increasingly diagnosed in recent years.

VAD may cause cerebral ischemia in about 80% of cases [3] and predominantly affect patients during their professional life. The functional outcome, however, seems to be usually good in the majority of cases but there is still a lack of knowledge about the natural history of VAD and uncertainty concerning the appropriate follow-up management. Because recurrence of stroke or dissection is very low despite a mostly lacking morphological artery recanalization and it is nearly limited to the first weeks after dissection, it was questioned by Leys and Debette (2006) [4] what are the appropriate clinical monitoring parameters for follow-up. They argued that a systematic follow-up of the vascular lesions may induce anxiety both in patients and physicians and lead to inappropriate treatments.

Previous VAD studies mainly focused on classical outcome endpoints such as mortality and recurrence rate. In recent years, however, patient-centered outcome measures such as quality of life (QOL) gained increasing importance. The health-related quality of life after VAD was examined in a standardized manner for the first time in only two published studies [5, 6] in the last decade. Although functional outcome was good in the majority of their cases, the surprising main finding was a bad quality of life, measured by Stroke Specific Quality Of Life scale (SS-QOL) [7] despite of good functional outcome, scored by modified Rankin Scale (mRS) [8], in about 15% [5] to 30% of cases [6].

This important discrepancy of QOL and functional outcome after VAD remained to be sufficiently explained. Furthermore, the study design was limited: Most data were retrospectively collected and cognitive variables not examined, for example. In general, various factors have been shown to influence the QOL of patients after stroke without dissection, including post-stroke anxiety [9], depression [10] and cognitive impairment [11]. When starting the present study, however, the knowledge about the putative contributing role of cognitive as well as psycho-affective factors to QOL in VAD patients was lacking. A better understanding of clinical courses and their affecting variables with special respect to the biopsychosocial model [12] seemed to be of great importance for the neurorehabilitation of such VAD patients in the future.

The aim of this study was therefore as follows: (1) to evaluate the characteristics of patients after VAD with special focus on those with bad quality of life despite good functional outcome in comparison with positive and negative control patients in a prospective comparative study design (2); to identify contributing factors to quality of life after VAD, considering neurological, cognitive, and psychological variables (3); to identify the predictive factors for quality of life after VAD.

## Methods

### Study design and participants

This exploratory study was carried out in the Neurological Department of the University-affiliated teaching hospital AGAPLESION Diakonieklinikum Rotenburg, Germany. All data were prospectively collected from consecutively recruited patients who were admitted to our stroke unit under the suspected diagnosis of an acute stroke between October 2010 and June 2013. Three cohorts of consecutive patients were included in the study for the purpose of comparison: group D (dissection) as the main group comprised patients with first-time spontaneous vertebral artery dissection (VAD) of at least one vertebral artery, group I (ischemia) as a positive control group consisted of patients with acute cerebral ischemia such as stroke or transient ischemic attack (TIA) of the posterior circulation due to any other cause than dissection, and group M (mimics) was chosen as a negative control group of stroke mimics of the posterior circulation.

The diagnosis of VAD was based on typical findings such as intramural hematoma on axial cervical MRI, or string sign or long tapering stenosis on computer tomography (CT) / MRI angiography in accordance to Rodallec et al. [13] and in the context of a fitting medical history, i.e. typical type and onset of symptoms. The type of cause for ischemia in the group I was categorized according to the TOAST criteria [14]. The inclusion criteria were (1) a reliable diagnosis, (2) age between 18 and 85 years, and (3)] medically stable psychological and physical condition for testing, i.e. language competence of fluency, cognitive screening score ≥ 25/30 by Mini-Mental State Examination (MMSE) [15] and no need for persistent clinical monitoring.

Exclusion criteria were as follows: (1) VAD due to severe trauma - in contrast to conventionally as spontaneous labeled dissection due to minor prior cervical trauma which should be better termed “mechanical trigger event” according to Engelter et al. (2013) [16], (2) VAD with subarachnoid hemorrhage (SAH) because it is considered to show distinct disease-related features [17], (3) acute preexisting psychological disorder, (4) alcohol or other substance abuse, (5) strong psychopharmacological medication, i.e. more than one drug, a medium or high dosage or signs of sedative or cognitive side effects, or (6) concurrent or preexisting CNS morbidity or damage apart from white matter lesions (WML) and “minor strokes” without any disabling, in particular cognitive preexisting deficit.

Participants were examined for psychological, cognitive and neurological variables with special reference for functional outcome as well as quality of life 6 months after the index event. The first data collection in each patient was performed at baseline (time point t1) in the acute phase in hospital after clinical stabilization: neuro-status at admission by National Institute of Health Stroke Scale (NIH-SS) score [18] according to patient’s records, neurostatus (mRS) and clinical data at baseline according to examination and interview, cognitive screening as well as extensive neuropsychological testing by battery at baseline, and administering self-rating protocols for symptoms of anxiety and depression pre-baseline as well as stroke-related QOL pre-baseline. The second data collection was achieved by written standardized, structured questionnaire at follow-up (time point t2) 6 months after the initial event. It comprised standardized self-rating protocols for neurostatus (mRS), stroke specific QOL and posttraumatic stress symptoms, and in addition open questions for the clinical course. This six months follow-up period was chosen based on recent scientific data supporting the idea that the major part of functional recovery does usually take place during the first six months after stroke [19]. Furthermore, reinfarction as a negative event or recanalization of the former dissected artery vessel as positive event is most probable during the same time period of first six months.

The study protocol was approved by the Local Ethics Committee of the University of Bremen. All participants have given written informed consent.

### Clinical and neurological assessment

Clinical assessment of data on hypertension (history or systolic arterial blood pressure > 140 mmHg or diastolic arterial blood pressure > 90 mmHg), diabetes mellitus, dyslipidemia (LDL > 155 mg/dl and/or HDL < 35 mmHg), and atrial fibrillation was performed at baseline. Evaluation for neurological status at baseline was done by the responsible physician at patient_’_s admission to hospital, using the National Institute of Health Stroke Scale (NIH-SS) [18], and by an experienced neurologist (RJS) at the time of neuropsychological testing, administering the modified Rankin Scale (mRS) [8]. The latter one was also used to determine the functional outcome at follow-up, filled out by the patient. The mRS is considered to be the worldwide most established functional outcome measure after stroke. It provides seven scoring levels as follows: (0) no symptoms at all, (1) no significant disability despite symptoms, (2) slight disability, (3) moderate disability, (4) moderately severe disability, (5) severe disability, and (6) dead. In accordance with common convention, mRS score of 0–2 reflects a good functional status meaning independency in daily living. Therefore, mRS scoring of 0–2 at follow-up was used as a good functional outcome.

### Psychological assessment

For the assessment of psychological variables several self-rating measurement tools were introduced: The German version of the Hospital Anxiety and Depression Scale (HADS) [20] was administered at baseline to determine the grade of symptoms of anxiety and depression in the week before baseline. A total score ≥ 11 out of 14 items corresponds to a pathological result, a score between 8 and 10 means a suspect result. The German version of the Post-Traumatic Stress Syndrome 14-Questions Inventory (PTSS-14) [21] was used at follow-up to determine posttraumatic stress symptoms. In contrast to the primary application of the English version to intensive care unit patients, the German version was recently validated for its use on a broader spectrum of patients [21]. Achievable are 14 to 98 points from 14 items. The higher the score the more probable is a posttraumatic disorder. The cut off in the German version is defined as a score of 40 points with a sensitivity of 82% and a good specificity of 92%.

### QOL measurement

The health-related quality of life regarding the week before baseline and at follow-up was assessed by the German version [22] of the Stroke Specific Quality Of Life Scale (SS-QOL) [7]. It contains 49 items belonging to 12 domains. It generates 12 domain-related scores and a total score. A summary score of ≥4.0 was considered to indicate good QOL, a score ≤ 3.9 bad QOL in accordance with Fisher et al. (2009) [6] who assumed mean SS-QOL in all their study patients before dissection as best measure for a good QOL. The use of the German version in this study was authorized by their authors (pers. communication).

### Neuropsychological assessment

For the neuropsychological testing at baseline the longer established Mini-Mental State Examination (MMSE) [15] and the more sensitive Montreal Cognitive Assessment (MoCA) [23] were performed as cognitive screening tests in their German versions. Maximum scores of 30 points in each of both tests correspond to an unimpaired cognition. For more extensive testing, a neuropsychological test battery was used with cognitive tasks of nine cognitive domains with possible reference to cognitive functions of the anatomical structures of the posterior circulation, in particular the cerebellum.

Apart from (1) the computer-based test battery for alertness, divided and selective attention (TAP) [24], all other tests were paper and pencil tests: (2) Trail Making Test (TMT A and B) for combined attention and executive function [25], (3) the Tower of London (TL-D) for executive function [26], (4) mental rotation (LPS 7) for visual-spatial function [27], (5) the Five-Point Test (5PT) for spatial-cognitive function [28], (6) the Regensburger Wortflüssigkeitstest (RWT) for verbal fluency [29], (7) Verbal Learning and Memory Test (VLMT) [30], (8) Block tapping (BT) for the visual digit span [31] and (9) the Finger Tapping Test (FTT) for the hand motor function [32]. Every pathological result, that is a value below one standard deviation, in one of the nine above mentioned cognitive function test domains was transposed to a cognitive composite score for each individual for the purpose of interindividual comparison. Adding one additional base point in all individuals our self-constructed cognitive composite score (CCS) showed a range from score 1 = normal to 10 = completely pathological.

### Neuroimaging examination

MRI of the brain was regularly performed as a standard procedure in patients with suspected stroke such as the participants in our study. Patients with suspected cervical artery dissection received additional angiography, mainly MRI angiography, if there has not been already evident cranial computer tomography or even conventional angiography. In addition to routine work a semiquantitative visual grading of white matter lesions (WML) from grade 0 = no lesions to grade III = severe and diffuse white matter lesions was performed in each patient according to the criteria defined by Fazekas et al. (1987) [33] and Wahlund et al. (2001) [34]. Furthermore, the extension of lesions by acute infarction in cases of group D and I was measured and categorized into either a maximal diameter > 10 mm or ≤ 10 mm.

### Statistical analysis

Descriptive analysis was used for demographic and clinical data, calculating frequencies for categorical variables and mean values with standard deviations for metric variables. Differences in baseline or follow-up characteristics between the three groups (D, I, M) were analyzed with Chi-square tests for categorical and with Kruskal-Wallis-test (H-test) for metric variables. In case of significant difference, a subsequent analysis between two group pairs was performed: For categorical variables the Chi-square test or Fishers exact test, if appropriate, and for metric variables the Mann-Whitney U-test was used with Bonferroni-correction of cumulative alpha-error. Subgroup-analyses were calculated for patients with mRS 0–2 and SS-QOL ≥ 4.0 versus those with mRS 0–2 and SS-QOL ≤ 3.9.

Follow-up group comparison of variables of paired samples for changes over time was performed by using the Wilcoxon-test. A Spearman’s rank correlation analysis was performed for outcome-relevant variables. By means of linear regression analysis, we first calculated the predictive value of certain variables for the variance of SS-QOL scores at follow-up in an univariate model. Afterwards we developed a multivariate regression model using variables that have proven statistically significant at the univariate analysis at a significant level of 5% (*p* < 0.05). The statistical data analyses were conducted using SPSS Statistics Version 22 and WinStat®, Bad Krozingen.

## Results

### Baseline data

**Fig. 1:**
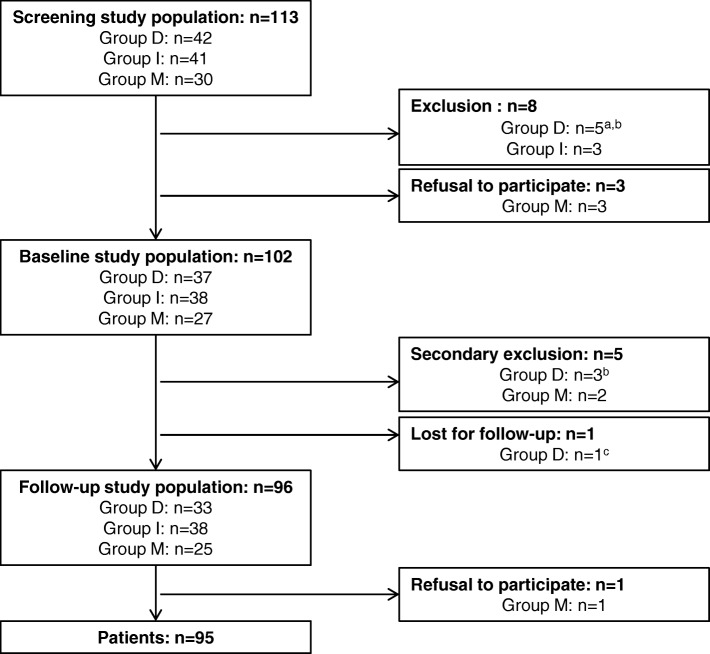
Flow diagram of the study population, ^a^ too severely disabled; ^b^ concurrent cerebral disease („dual pathology“); ^c^ deceased

From October 1, 2010, to June 31, 2013, 42 consecutive patients with spontaneous first-time vertebral artery dissection (VAD), who were admitted to our hospital under the suspected diagnosis of an acute stroke, were registered and screened for this study. 37 patients fulfilled the inclusion criteria and were included into the study as group D. In addition, 38 patients with acute stroke or TIA of the posterior circulation were included as comparison group I and 27 stroke mimics of the posterior circulation as comparison group M (Fig. 1). Secondary exclusion due to defined criteria decreased the number of baseline patients of group D to 34 and group M to 25.

Five patients were excluded from the dissection study group after screening according to the inclusion/exclusion study criteria: One woman (70 years old; ataxia, dizziness, facial weakness; medulla oblongata infarction by vertebral artery occlusion suspicious for but not yet proven dissection) deceased due to unexpected cardiopulmonary failure not otherwise specified in the acute phase. Another woman (47 years old; locked in-syndrome due to pontine infarction by basilar artery occlusion due to vertebral artery dissection) and a man (77 years old; dizziness, nausea, vomiting, headache, ataxia, facial weakness, dysarthria, dysphagia; combined medulla oblongata and cerebellar infarction) without a sufficient ability to speak for participating in neuropsychological testing. Two more men (46 and 56 years old; one with bilateral embolic cerebellar and occipital brain infarctions by VAD plus ocular down-beat syndrome, unilateral motor dysfunction, dizziness, and headache; the other one with dizziness and ataxia due to suspected cerebral ischemia by VAD) wanted to cut their inpatient treatment short and not to participate but showed also contraindications in form of concurrent diseases: one developed bronchial carcinoma 4 months later and brain metastases 8 months later, then deceased, the other suffered from alcoholism and had a history of former bronchial carcinoma and prophylactic brain irradiation.

Three initially included woman of ages 70, 71, and 77 years had to be excluded secondarily because of concurrent diseases in form of preexisting idiopathic cerebellar syndrome, acute symptomatic anterior circulation brain infarction, and predominant arteriitis temporalis (first with ocular disorder, paresis of arm, ataxia, and dysarthria plus suspected brainstem infarction due to VAD; second with VAD and arm paresis; third with VAD and visual field disorder).

Types of stroke mimics were predominantly disorders of the vestibular system with vertigo or dizziness as main symptoms. They included benign paroxysmal positional vertigo in 40%, vestibular neuritis in 24%, vestibulocochlear irritation in 4%, Schwannoma in 4%, suspected somatoform dizziness in 8% and nonspecific dizziness of unknown origin in 16%. Apart from the presence of ischemic stroke lesions in groups D and I, socio-demographic and clinical data of the three groups were without significant differences, as shown in Table 1.

**Table 1:**
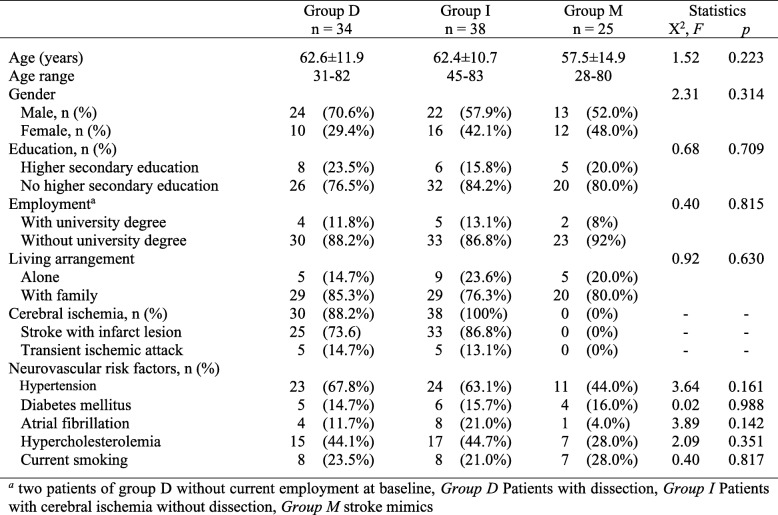
Demographic and clinical characteristics at hospital admission

VAD affected the right side in 13 patients (38.2%), the left side in 18 patients (53%), and both sides in three patients (8.8%). The majority of dissected arteries showed (subtotal) occlusion (*n* = 25; 67.6%) or stenosis (*n* = 8; 21.6%), the remaining ones no stenosis at all (*n* = 4, 10.8%). Twenty-three patients with VAD had (subtotal) occlusion, eight patients a stenosis and three no significant stenosis. Three out of 34 showed dissected vertebral arteries on both sides (two patients with bilateral occlusion each, one patient without any stenosis).

Sixteen patients (47%) presented with vertigo or dizziness as either the only symptom or among other symptoms. 88.2% of patients with dissection (group D) experienced acute cerebral ischemia. They showed ischemic stroke lesions in the majority of cases (73.6%) like the patients of group I (86.8%), larger than 10 mm in maximal diameter in 67.6% versus 76.3%, respectively, as shown in Table 2. Cerebral ischemia in group I patients was caused by cardiac embolism in 34.2%, lacunar disease in 21%, large artery disease in 10.5% and by undetermined cause in 34.2% according to the TOAST-criteria [14].

**Table 2:**
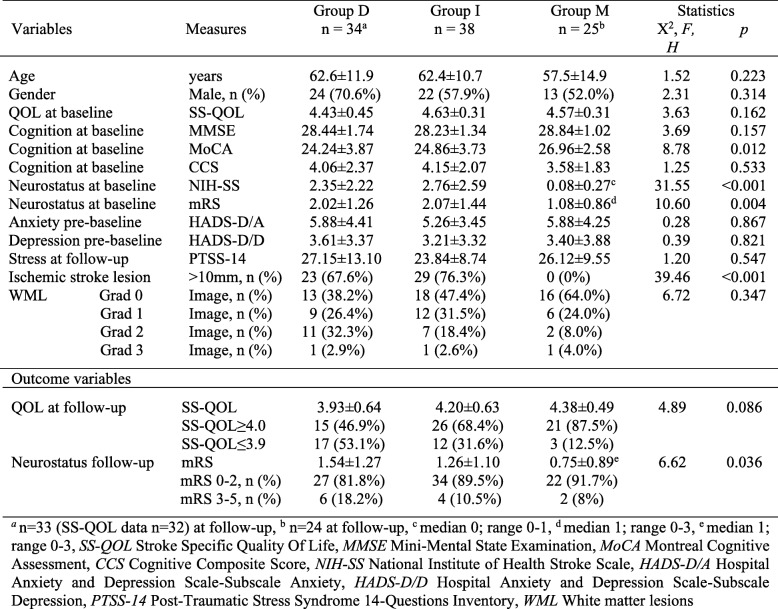
Contributing factors to quality of life at follow-up (above) and outcome variables (below)

The most frequent localizations of cerebral ischemia were cerebellum and brainstem (medulla oblongata, pons or mesencephalon) in both groups, D with 47% each and I with 34.2 and 42.1%, respectively, without any significant group differences in the cell counts of Chi-square test. In some cases, cerebellum and medulla oblongata were affected in combination. Other sites of ischemia were occipital lobe in 17.6% of group D and 34.2% of group I, furthermore thalamus in 5.3% of group I. The grade of white matter lesions (WML) showed no significant difference between groups, even if it was less frequent in stroke mimics. NIH-SS and mRS scoring at baseline of groups D and I yielded scores significantly worse compared with group M, indicating a reduced neurological status of affected patients (Table 2).

Comparison of cognitive baseline profiles of the three groups revealed no significant differences. While there were in groups D and I compared to group M frequent impairments in both global screening tests (MMSE, MoCA) and in single cognitive function tests of the neuropsychological test battery (Additional file 1) and also in the resulting cognitive composite score (CCS), this difference reached statistical significance only in the MoCA assessment for group D compared to group M (Table 2). Trends towards worse cognitive function without statistical significance were found in group D and I compared to group M for the following cognitive domains: divided and selective attention (TAP), combined attention and executive function (TMT A and B), mental rotation (LPS-7), and spatial cognitive function (FPT). Spearman’s rank correlation analysis yielded a highly significant correlation (*p* < 0.001) between all three cognitive scoring systems (MMSE, MoCA, and CCS).

Regarding premorbid psychological profiles, no significant group differences of the mean values of scoring systems (HADS-A/D) for symptoms of anxiety or depression in the week before baseline were found. Likewise, there was no statistical group difference of the mean values of the total quality of life score measured by SS-QOL. Group D only displayed a significant lower mean value than groups I and M in the domain “social roles”. No further differences were found in the other domains.

### Clinical courses and follow-up: functional outcome, QOL and stress symptoms

There was a high responder rate in follow-up assessments: **f**ollow-up data were obtained in 97% of patients in group D, 100% in group I, and 96% in group M. **S**econdary ischemia prevention by medication until follow-up was provided by platelet aggregation inhibitors in 76.5% patients of group D and 81.6% of group I and oral anticoagulants in 23.5% patients of group D and 18.4% of group I. Vascular events of importance occurred until follow-up time point as follows: In group D one recurrent stroke, one suspected stroke, one new stroke due to dissection of the internal carotid artery, and one transient ischemic attack. Another 66 years old patient with medulla oblongata infarction deceased due to nonspecific heart failure and was lost for follow-up; in group I one stroke and two myocardial infarcts occured; group M remained without any vascular event. Important non-vascular incidents were a newly diagnosed prostate cancer with radiation therapy in group D and an inpatient treatment for depression in one patient as well as a single epileptic seizure in another one in group I. Three patients of group M suffered from recurrent benign paroxysmal positional vertigo, new vestibular neuritis and nonspecific dizziness of unknown origin, respectively.

**Fig. 2:**
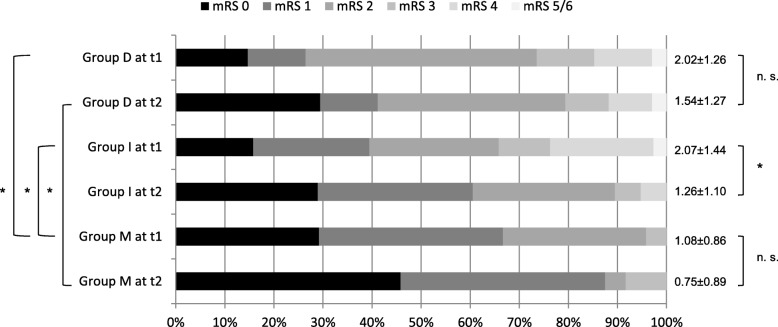
Group-related changing in mRS scoring between time points t1 (baseline) and t2 (6 months follow-up), *group D* patients with dissection; *group I* patients with ischemia without dissection; *group M* stroke mimics; *mRS* modified Rankin Scale; *n. s.* not significant, * significant difference

Inpatient rehabilitation for several weeks was performed in 48.5% patients of group D, 60.5% of group I and in only one patient (4%) of group M. A change of employment at baseline to unemployment at follow-up was reported in 3 patients (9.4%) in group D, 5 patients (13.5%) in group I and 2 patients in group M. Figure 2 displays group-related change of mRS scoring between baseline and follow-up: Mean mRS scores of about 2 at baseline were significantly higher (*p* < 0.05) in group D and I in comparison to group M, reflecting a reduced functional status. Mean values of mRS scoring improved from baseline to follow-up in all three groups but significantly only in group I. At follow-up assessment, group M showed the best distribution of mRS scores in direction to better ones and group D the worst with a significant higher mean score of mRS.

Most patients achieved good QOL (SS-QOL ≥ 4.0) at 6 months follow-up in group I (68.4%) and even better in group M (87.5%) in contrast with group D (46.9%) (Table 2). Intergroup analysis of change of mean total scores of QOL, as measured by SS-QOL, from pre-baseline to follow-up displayed a significant deterioration (*p* < 0.001; Wilcoxon signed-rank test) in groups D and I but not in group M. Further analysis of changing scores in the twelve SS-QOL domains yielded developing impairments from baseline to follow-up in all three groups D, I, and M. Impairments evolved mainly in psychosocial domains such as “family roles”, “social roles” and “energy” and less in physical domains. Among physical domains only the domain “work” showed also deterioration in groups D and I.

Study participants were asked at follow-up to answer 14 items of the PTSS-14 inventory regarding stress symptoms in the previous week. A total score of ≥40 points may be indicative for posttraumatic stress disorder. Group-related mean values were below this cut-off level and not significantly different between groups: group D 27.15 ± 13.10, group I 23.84 ± 8.74, and group M 26.12 ± 9.55. In five patients (15.1%) of group D, however, PTSS-14 scoring was > 40, indicating possible posttraumatic stress disorder.

### Subgroup analysis

SS-QOL scores at follow-up varied among subgroups as demonstrated in a subgroup analysis stratified for good functional outcome (mRS 0–2) plus good quality of life (SS-QOL ≥ 4.0) versus good functional outcome (mRS 0–2) plus bad quality of life (SS-QOL ≤ 3.9). Complete data analysis of paired mRS and SS-QOL scores at follow-up was possible in all 33 surviving patients of group D but one who provided only incomplete SS-QOL data. Thirteen of these patients (40.6%) showed a bad quality of life (SS-QOL ≤ 3.9) despite good functional outcome (mRS 0–2) and likewise thirteen (40.6%) a good quality of life (SS-QOL ≥ 4.0) combined with a good functional outcome (mRS 0–2). In group I 26.3% of all patients displayed mRS 0–2 and bad quality of life (SS-QOL ≤ 3.9), whereas 63.1% mRS 0–2 and good quality of life (SS-QOL ≥ 4.0). Among 24 stroke mimics only two patients (8.3%) with mRS 0–2 reported a bad quality of life in contrast to 20 patients (83.3%) with mRS 0–2.

In the SSQOL-subgroup analysis of patients with good functional outcome (mRS score ≤ 2) and good SS-QOL score (≥4.0) at follow-up were eight patients with arterial occlusion or subtotal occlusion versus five with or without stenosis. The subgroup of patients with good functional outcome (mRS score ≤ 2) and bad SS-QOL score (≤3.9) comprised ten patients with arterial occlusion or subtotal occlusion versus three with or without stenosis. This difference was not statistically significant (*p* < 0.05).

**Fig. 3:**
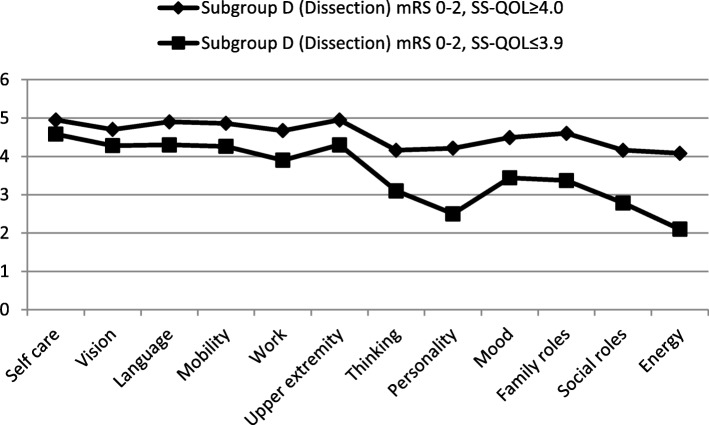
SS-QOL-subgroup analysis of patients with dissection and mRS score 0–2 at follow-up, *SS-QOL* Stroke Specific Quality Of Life; *mRS* modified Rankin Scale

Analysis of subscales demonstrated that a reduced quality of life at follow-up (SS-QOL ≤ 3.9) in both subgroups (mRS 0–2) of group D and I corresponded to main impairments, that were significantly reduced mean values, in all psychosocial domains such as “Thinking”, “Personality”, “Mood”, “Family Roles”, “Social Roles” and “Energy”, as shown for subgroup D in Fig. 3, in comparison to good outcome subgroups of D and I with mRS 0–2 and SS-QOL ≥ 4.0. Further subgroup analysis was performed in subgroups D and I by comparison of variables potentially affecting quality of life such as age, gender, neurocognition at baseline (MMSE, MoCA), neurostatus at baseline (NIH-SS), grade of white matter lesions (WML), burden of ischemic stroke lesions, premorbid (pre-baseline) symptoms of anxiety or depression (HADS), stress symptoms at follow-up (PTSS-14), and extent of decrease of QOL from pre-baseline to follow-up (Table 3).

**Table 3:**
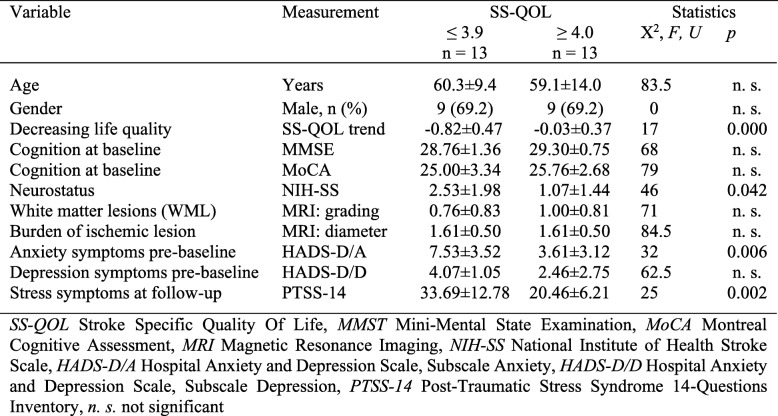
Comparison of subgroup D patients with mRS 0–2 at follow-up: SS-QOL ≤ 3.9 vs. SS-QOL ≥ 4.0

The mean decrease of QOL, that is the difference of SS-QOL scoring, from pre-baseline to follow-up, was significantly stronger in the subgroups (mRS 0–2) with bad quality of life (SS-QOL ≤ 3.9). The main findings were found in subgroup D (mRS 0–2) with bad QOL ≤ 3.9 that were significantly higher mean values for premorbid anxiety symptoms (*p* = 0.006) and stress symptoms at follow-up (*p* = 0.002). Other important findings were found in subgroup I (mRS0–2) with bad QOL that were significantly higher mean values for premorbid anxiety symptoms (*p* = 0.002) and depression symptoms (*p* < 0.001). Differences in reduced neurostatus at baseline (NIH-SS on admission)(*p* = 0.042) in subgroup D (mRS 0–2) SS-QOL ≤ 3.9 vs. SS-QOL ≥ 4.0 and higher grade of white matter lesions (WML)(*p* = 0.042) in subgroup I (mRS 0–2) SS-QOL ≤ 3.9 vs. SS-QOL ≥ 4.0 were not significant after correction of cumulative alpha-error. The other variables showed no significant differences.

Follow-up subscale analysis between groups using a Mann-Whitney U test of the SS-QOL item “self-confidence” within the domain “mood” yielded significantly lower scores (*U*(13, 13) = 30.5; *p* < 0.01) for VAD patients with bad QOL (SS-QOL ≤ 3.9) versus good and also significantly lower scores (*U*(9, 25) = 24.5; *p* < 0.001) for group I patients with bad versus good QOL.

### Predictors of QOL

Preceding correlation analyses in this study yielded significant correlations in between all neurocognitive measures such as MMSE, MoCA, and CCS at baseline and likewise in between both neurostatus measures such as NIH-SS on admission and mRS at baseline. Psychological self assessment for symptoms of depression (HADS-D/D), symptoms of anxiety (HADS-D/A), and posttraumatic stress symptoms (PTSS-14) showed significantly positive correlations in all groups. Impairments in neurocognitive screening tests (MMSE, MoCA) correlated weakly with neurological impairments as measured by NIH-SS. MMSE and MoCA inversely cross-correlated with NIH-SS scores in group D with weak significance, MoCA scoring with NIH-SS also significantly in group I, and MMSE scoring with NIH-SS only non-significantly in group I.

All groups (D, I, and M) displayed a significant correlation between age and white matter lesions (WML). In addition, the extent of WML showed a significant inverse correlation to global cognitive functioning (MMSE, MoCA) in group D and partly (MoCA only) in group M. Finally, more extensive stroke lesions correlated very weakly with stronger neurological impairment (higher NIH-SS score at baseline) in group D and showed no other consistent correlations.

Table 4 shows univariate linear regression analysis demonstrating that neurocognition scores at baseline (MMSE, MoCA, CCS), neurostatus at baseline (NIH-SS score on admission, mRS score) and stress symptoms at follow-up (PTSS-14 score) were predictors of quality of life at follow-up. In a subsequent multiple regression analysis, neurocognition at baseline measured by MMSE, neurostatus at baseline measured by mRS and posttraumatic stress symptoms measured by PTSS-14 proved to be independent predictors for the quality of life at follow-up, explaining in combination 71% of its variance**.** Thus, reduced neurocognition and neurostatus at baseline and increased scoring levels for stress symptoms at follow-up were predictive for reduced quality of life at follow-up in patients with VAD in this regression model.

**Table 4:**
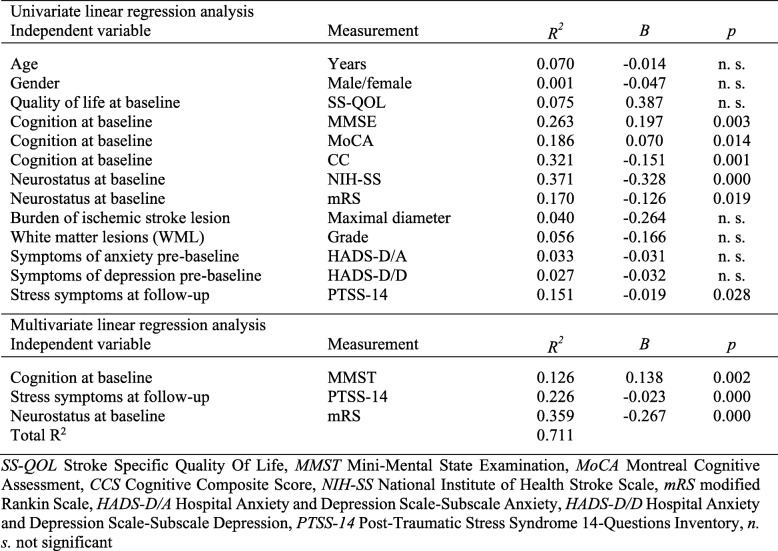
Regression analysis of predictors for SS-QOL at follow- up in patients with dissection

## Discussion

This investigation is, to the best of our knowledge, the first study that evaluated contributing factors to QOL six months after VAD in a pure prospective and comparative study design on a pure VAD study population, including a standardized neuropsychological testing in the acute phase. We found reduced QOL at 6-month follow-up, as scored by SS-QOL ≤ 3.9, despite good functional outcome (mRS 0–2) in a prevalently high percentage of about 40% in VAD patients. The variance of total QOL was determined by neurological, neurocognitive and psychological predictive factors. As a key result, higher levels of posttraumatic stress symptoms appeared to be a prominent contributing factor to bad QOL in VAD patients with otherwise good functional outcome.

Cross sectional analysis of our baseline data displayed no significant group differences regarding sociodemographic variables. Likewise, mean values of pre-baseline total QOL were not significantly different. Although there was a preponderance of atrial fibrillation in group I, no significant group differences of neurovascular risk factors were found in line with current knowledge about VAD characteristics [3, 35].

The prevalence of 73.6% VAD patients with ischemic stroke and 14.7% with TIA in our study corresponded well to 67% (114 patients) and 10% (17 patients), respectively, in a large European multicenter prospective study on patients with first-ever spontaneous VAD [3]. In line with previously published and reviewed data [36], the rate of ischemic recurrence of 9% in our VAD patients (literature estimates between 0 and 13.3%) and rate of recurrent dissection of 3% (literature estimates between 0 and 25.0%) were low. Likewise, in concordance with the literature [5, 6, 36], functional outcome was good (mRS0–2) in the majority of patients (81.25%). While SS-QOL at follow-up was normal and corresponded to pre-baseline in stroke mimics, SS-QOL scores significantly worsened in group D and I patients, mainly in the psychosocial domains.

Published data about the possible impact of stenosis or occlusion of dissected vertebral artery to outcome have been rare and inconsistent so far. The multivariate analysis of 126 patients with carotid and vertebral artery dissections in a retrospective study design showed that the variables stroke and arterial occlusion were independent factors associated with a poor outcome [37]. Traenka et al. (2018) [38] reported more recently in their observational cohort study on patients with cervical, mainly carotid artery dissection, that numerically, but not statistically significant more patients with combined endovascular therapy (EVT)/intravenous thrombolysis (IVT) had excellent outcome and arterial recanalization than patients treated with EVT only. In our study, there was a trend in patients with good functional outcome (mRS score 0–2) to higher association of occlusion/subtotal occlusion with worse QOL, though this was statistically nonsignificant (*p* < 0.05).

Consequently, a valid and reliable evaluation of this variable as putative contributing factor appeared to be not adequately possible in our study: Besides the sample size being very small, it remains unclear how to operationalize best the vascular measurements such as (1) determination of grade/severity of stenosis and by which method (MRI or ultrasound), (2) length of stenosis or occlusion, or (3) site of stenosis (unilateral left or right, bilateral, additionally extra-vertebral). Furthermore, some recently published data suggested for the first time a role of VAD-accompanied atherosclerosis as additional putative contributing factor to functional outcome at three months in posterior circulation stroke (PCS) patients [39]. This may also refer to some of the older patients in our study though we have not examined them for arteriosclerosis in such detail. The method of evaluation, i.e. grading of atherosclerosis and at which site, has to be further clarified and addressed by future studies of larger sample volumes.

Because previous studies have shown no crucial role of neurovascular risk factors or sociodemographic factors for QOL of VAD patients, we focused our analysis of potential contributing factors for QOL on previously less investigated neurological, neurocognitive and psychological variables with special respect to the biopsychosocial model [12]. Psychological variables such as pre-baseline symptoms of anxiety and depression were not significantly different between our groups. Furthermore, by multiple regression analysis, they were no predictors of QOL variance of VAD patients at follow-up.

There were significant more neurological impairments at baseline, that are higher scores of mRS for functional disability or NIH-SS for neurological deficit, in group D as well as I patients with ischemic stroke lesions compared to stroke mimics. Furthermore, as plausible finding, NIH-SS and mRS scoring at baseline showed a significant positive correlation in our study. Likewise, functional impairment measured by mRS at follow-up significantly correlated with reduced SS-QOL at follow-up in concordance with the results of the mixed cervical artery dissection series of Fischer et al. (2009) [6]. Corresponding to their multivariate analysis, the NIH-SS score on admission was also found to be an independent predictor of QOL at follow-up in our univariate regression analysis. Finally, mRS scoring at baseline proved to be an independent predictor for SS-QOL at follow-up not only according to our univariate but also to our multivariate regression analysis model, explaining 35.9% of QOL variance according to our regression model.

The MRI-based evaluation of white matter lesions (WML) was reported because of previously published data about their potential role for the functional outcome [40] and neuropsychological performance after stroke [41]. Kissela et al. (2009) [40] reported that severe periventricular white matter disease was significantly associated with poor functional outcome at 3 months after ischemic stroke, independently of other factors. Jokinen et al. (2005) [41], for example, noted a correlation of the degree of WML with cognitive decline. Other published data remained inconsistent. Even if WML predominated in groups D and I compared to stroke mimics, they showed no significant inter-group difference. Moreover, our data demonstrated no role of WML as independent predictor of QOL.

Because data on the potential impact of infarct volume to outcome and QOL in VAD patients have been lacking so far, we used at least a very arbitrary method for semiquantitative evaluation of the extension of infarct lesions and were not able to ascertain any statistical association. Apart from the limits of our measurement method, we assumed that the neuroanatomical function of the affected stroke area was much more important than the extension.

The finding of more neurocognitive impairments in form of lower mean values in cognitive measures in patients with ischemic lesions of both group D and group I patients did not reach significance. Previous studies described poststroke cognitive decline by global cognitive screening such as MMSE and more recently and more sensitively by MoCA [42]. MoCA, to our best knowledge, was used in our study for the first time in VAD patients. While the mean group values of MMSE around 28 out of 30 were within normal range, only the group of stroke mimics showed a normal mean value of 26.96 if scored by MoCA. Mean MoCA values of group D (24.24) and I (24.86), however, displayed slightly pathological scores. They most probably reflected stroke lesion-associated cognitive impairments whereas stroke mimics without any lesions did show normal scores. Lower scores of both global screening systems, MMSE and MoCA, were independent negative predictors for QOL at follow-up in univariate regression analysis. In multivariate regression analysis only MMSE remained a poor predictor for QOL, explaining 12.6% of its variance.

Although MMSE and MoCA significantly correlated to our cognitive composite score (CCS), further analysis of neurocognitive domain deficits by neuropsychological test battery yielded only some trends of mean group values, without statistical significance. The mean group values of single tests as well as of CCS showed at least clear trends of stronger cognitive impairments in group D and group I patients than stroke mimics regarding the following cognitive domains: Divided and selective attention (TAP), combined attention and executive function (TMT A and B), mental rotation (LPS-7), and spatial cognitive function (FPT).

These findings corresponded widely to findings of Gottwald et al. [43] who preoperatively examined patients with cerebellar hematomas or brain tumors by the same neuropsychological tests apart from not using LPS as we did. This profile of cognitive dysfunction was related to predominance of cerebellar stroke lesions in both group D and group I. The findings were in line with modern concepts of cerebellar cognitive function [44] and also in accordance to previous data on cognitive impairments in patients with cerebellar stroke lesions, for example by Exner et al. (2004) [45]. Speck et al. (2014) [46] were the only other ones to date who recently published cognitive status data of patients after cervical artery dissection. Their mixed series included about two third of patients with spontaneous internal carotid artery dissection (ICAD) and one third with VAD. Ischemic stroke was found in only 33.9%. Thirty-one of 62 study participants completed 18.9 ± 22.72 months after discharge four tests of attention and memory function. Because only three of them showed any signs of cognitive impairment, the authors argued that deficits were unlikely responsible for the reduced QOL.

Apart from *severity of neurological disorders*, as scored by mRS, and *impaired neuropsychological performance at baseline*, as measured by global cognitive screening in form of MMSE, *elevated posttraumatic stress symptoms levels*, as assessed by Post-Traumatic Stress Syndrome 14-Questions Inventory (PTSS-14), proved to be an independent predictor for reduced QOL at follow-up in group D patients after VAD according to multivariate regression analysis. The PTSS-14 was developed by Twigg et al. (2008) [47] in the United Kingdom (UK) as a new, more practical screening tool for post-traumatic stress disorder (PTSD). It showed a high validity [47] against the Posttraumatic Diagnostic Scale (PDS) [48] as longer established 49-item self-report measure. While UK-PTSS-14 was initially applied to patients after intensive care unit (ICU) discharge, Radtke et al. [21] broadened its application when evaluating the validity of the German version, showing a sensitivity of 82% and specificity of 92%. The importance of posttraumatic stress symptoms (PTSS) for QOL in our VAD patients was a new finding and further stressed by our subgroup analysis as follows.

One main finding was the high prevalence of reduced QOL despite good mRS (0–2) in about 40% (*n* = 13) of our VAD patients at follow-up in line with the findings of the observational series Czechowsky et al. (2002) [5] and Fischer et al. (2009) [6]. Czechowsky et al. (2002) [5] obtained 0.3–3.8 years after VAD follow-up data in 21 surviving patients who were retrospectively contacted. They found 81% with good functional outcome (mRS0–2) but only 66.6% with good SS-QOL scoring. Fischer et al. (2009) [6] prospectively found 379–3455 days after event 30% patients with impaired SS-QOL scoring among 66% with favorable functional outcome (mRS0–1) in a mixed series including patients with VAD and patients with ICAD.

As the key finding, our subgroup analysis of these VAD patients showed significantly higher levels of posttraumatic stress symptoms (*p* = 0.002) and of pre-baseline anxiety symptoms (*p* = 0.006) being associated with patients with good functional outcome (mRS0–2) and bad QOL compared to those with good functional outcome (mRS 0–2) and good QOL. Nearly all other potential contributing factors were not significantly different. Elevated levels of posttraumatic stress symptoms, as evaluated by PTSS-14 scoring in our study, are in line with recent reports on the prevalence of posttraumatic stress disorder (PTSD) after stroke, even after minor stroke [49] or transient ischemic attack [50]. While post-stroke anxiety [9] and depression [10] have been described already earlier, even depression in stroke patients treated and non-treated with intravenous thrombolytic therapy [51], posttraumatic stress disorder has been coming to attention more recently.

Speck et al. (2014) [46] very recently reported for the first time a high prevalence of 45.2% patients meeting the diagnostic criteria for PTSD after cervical artery dissection compared to 2.9% in the general German population. They assessed, partly retrospectively two months to five years, partly prospectively one month after dissection, the presence of PTSD by using the Posttraumatic Diagnostic Scale (PDS) as self-rating questionnaire. Their series comprised physically less affected patients, two-third after ICAD and one-third after VAD, with ischemic stroke in form of mainly small lesions in about one-third of cases only. Their high PTSD prevalence might be overestimated because physically less affected people voluntarily participating in their study might have tended to mention more mental problems when asked by self-rating PDS. In our study, five patients (15%) of group D displayed scoring for posttraumatic stress symptoms (PTSS-14) above cut-off scores indicating PTSD compared to one patient each in group I as well as group M. Previous data [52], however, have already stressed that even subsyndromal scores may be of relevance, as can be assumed for our thirteen subgroup D patients with elevated PTSS-14 levels and bad QOL despite good functional outcome.

The elevated levels of stress symptoms after VAD in our study may be interpreted as maladaptive psychological state/condition. In this context the following aspects seem to be worthy to note: First, elevated scores of stress symptoms were also found in patients without any stroke lesion in our study in line with other study results [50]. Second, apart from elevated scores of stress symptoms, significantly higher scores of pre-baseline symptoms of anxiety were found which might be indicative for a predisposing vulnerability for anxiety disorders and subtypes like (subthreshold) posttraumatic stress disorder. Third, apart from elevated scores of stress symptoms significantly lower scores of the SS-QOL item “self-confidence” within the domain “mood” were found at follow-up. Fourth, maladaptive coping strategies were significant predictors for and associated with posttraumatic stress disorder in patients with cervical artery dissection in the study of Speck et al. (2014) [46]. We think increased PTSS levels were neither decisively stroke unit-related, as they were less frequent in comparison group I and M patients who were also treated on the stroke unit, nor disease-specific, as they were also present in group I and M.

PTSS levels have been still prevalent in group D which might be explained by the stress-vulnerability model [53]. According to modern stress concepts, situations that in particular include unpredictability and uncontrollability can trigger stress [54]. Both conditions fit if someone is suffering from a spontaneous artery dissection as well as subarachnoid hemorrhage in contrast to ischemic stroke caused by vascular risk factors which can be treated. Noble et al. (2008) [55], for example, prospectively studied 105 subarachnoid hemorrhage patients at 3 and 13 months post-ictus and found that 37 % met the diagnostic criteria of PTSD. Furthermore, PTSD was the single best predictor of patients’ mental QOL in their study.

Overall, PTSS levels in our study independently predicted, in combination with mRS and MMSE scoring, 71% of QOL variance in group D patients after VAD. Furthermore, it may sufficiently explain the predominant impairments of psychosocial QOL domains after VAD. It has been already earlier demonstrated in both patients with stroke and patients with Parkinson’s disease that the type of psychosocial alterations, psychosocial adaptation and coping strategies seem to be of much greater impact than the degree of physical impairment [56].

Importantly, our study cohort of VAD patients appeared to be not significantly biased by exclusion of eight patients, whose features and mean age of 64.5 years widely resembled those of the study group. More importantly, however, our study showed also several limitations. First of all, the study cohort showed an unusual high percentage of elderly VAD patients (mean age 62.6 ± 11.9). They were almost 20 years older than in other large study samples ( [3], mean age 43 ± 9 [57], mean age 41.1 ± 9.9]. Cervical artery dissection is commonly considered to be underdiagnosed [3, 57–59]. Grond-Ginsbach et al. (2013) [59] hypothesized that cervical artery dissection goes frequently undiagnosed, particularly in patients with subtle symptoms, which is true for VAD [3, 57], and dependently from the awareness of the responsible physician. Furthermore, cervical artery dissections in patients ≥60 years are often painless and mechanical triggers missing [60]. In addition, differentiation of dissection from rupture of atheroma in the context of arteriosclerosis may be difficult so that Ahl et al. (2004) [58] proposed the term of “atherosclerotic dissection” for certain cases.

Over the last years, cervical artery dissection has been increasingly diagnosed due to improved neuroimaging methods [58]. That is in particular striking regarding VAD. Ahl et al. (2004) [58] showed in their study that a significant number of cervical artery dissection can occur in the older age group and can be diagnosed if considered. They finally hypothesized that the rate of incidence must be equal throughout life. The overrepresentation of elderly patients in our study may be most probably explained to some extent by a hospital-based selection bias: (1) Patients were recruited when referred to the supraregional stroke unit of our teaching hospital. (2) Broad neuroimaging of cervical arteries including cervical MRA was regularly applied to patients of all age groups and risk profiles whenever differential diagnosis of VAD appeared to be possible. (3) The upper inclusion limit of age range for our study was quite high with 85 years.

Despite obvious overrepresentation of elderly VAD patients and the need for confirmation of our results by larger studies, the main findings are in line with all three younger aged study cohorts that were previously examined for health related QOL after cervical artery dissection as discussed above: (1) Significant percentage of patients with bad QOL (SS-QOL scoring) despite good functional outcome (mRS sccoring) - Fischer et al. (2009) [6], mean age 46 years, Czechowsky et al. (2002) [5], mean age 50 years, (2) posttraumatic stress symptoms as significant predictor for reduced SS-QOL – Speck et al. (2014) [46], mean age 44.8 years. In addition, the autopsy findings of the 66 years old patient with medulla oblongata infarction by VAD who deceased due to unexpected heart failure after baseline confirmed the diagnosis of VAD and support the validity of our data.

The senior age of VAD patients may have several important implications for social life. In contrast to younger people who are supported by their also young family as well as the health system to achieve occupational reintegration as fast as possible, elderly people may be limited by less easy access to rehabilitation facilities, less support by potentially also disabled caregivers and, probably most important, by potential comorbidities and/or neurovascular risk factors. Knecht et al. (2015) [61] reported that older stroke patients in general have worse prestroke status, greater impairment on hospital admission, more comorbidities and poorer poststroke functional status than the younger patients but can benefit as much as the young from high-intensity neurorehabilitation. The challenging main consequence from our study regarding this older age group of VAD patients might be therefore for the treating physician to make the right decision: When to consider (re-)dissection ± stroke and when to consider neuropsychiatric sequelae, for example. Thus, somewhat unspecific symptoms in this age group and context of VAD history, if not critically reflected, may usually prompt otherwise evitable inpatient diagnostics for stroke and/or recurrence of dissection.

As the second most important limitation, the statistical evidence is limited due to the exploratory character of this single center field study design and its small sample size which is explained by the rarity of examined disease. Thus, the present investigation has to be characterized as an explorative study. Third, psychological condition was not examined at baseline. It was only asked for affective symptoms of anxiety and depression pre-baseline and at follow-up there has been only exploration for stress symptoms but not for symptoms of anxiety and depression. Furthermore, there was no physical follow-up examination conducted, only a follow-up assessment by questionnaire. Neurocognitive domain assessment at baseline was dichotomized into normal versus pathological values based on a difference of more than one standard deviation. Therefore, pathological alterations might have been either too subtle for detection, the applied neuropsychological tests not sensitive enough, or the study cohorts too heterogenous. Finally, additional potential outcome-relevant lifestyle-factors such as nutrition and sports activity as well as social factors such as social networks and social support were not taken into account of this study.

## Conclusions

In conclusion, VAD may significantly impair QOL at 6 months follow-up by multiple factors. It leads to a reduced QOL in a significant percentage of patients despite good functional outcome. Our data suggest that posttraumatic stress symptoms are of significant importance for the QOL after VAD, in particular in patients with reduced QOL despite good functional outcome. For both future research and clinical treatment, our data favor a multidimensional monitoring after VAD, with special focus on neuropsychiatric sequelae. Psychometric self-rating tools, as used in this study, may enable timely detection of such sequelae and facilitate therapeutic intervention.

## Supplementary information

**Additional file 1.** Data about pathological results of neuropsychological test battery assessment.

## Data Availability

The datasets used and analysed during the current study are available from the corresponding author on reasonable request.

## Abbreviations

5PT: Five-point test
BT: Block tapping
CT: Computer tomography
D: Dissection
FTT: Finger tapping test
HADS: Hospital anxiety and depression scale
HDL: High density lipoprotein
I: Ischemia
ICAD: Internal carotid artery dissection
LDL: Low density lipoprotein
LPS: Leistungsprüfsystem
MMSE: Mini-mental state examination
MoCA: Montreal cognitive assessment
MRI: Magnetic resonance imaging
mRS: Modified Rankin scale
NIH-SS: National Institute of Health Stroke Scale
PTSS-14: Posttrauamtic stress syndrome 14-questions inventory
QOL: Quality of life
RWT: Regensburger Wortflüssigkeitstest
SS-QOL: Stroke specific quality of life scale
TIA: Transient ischemic attack
TL: Tower of London
TMT: Trail making test
VAD: Vertebral artery dissection
VLMT: Verbal learning and memory test
WML: White matter lesions
