# Supplementary material for: Contributing factors to quality of life after vertebral artery dissection: a prospective comparative study
Source: BMC Neurol. 2019 Dec 4;19:312. doi: 10.1186/s12883-019-1541-x (PMC6894297; doi:10.1186/s12883-019-1541-x)
Supplement: Supplementary file 1 — Additional file 1. Data about pathological results of neuropsychological test battery assessment. [file 12883_2019_1541_MOESM1_ESM.pdf]

Table 5 Pathological results of the neuropsychological test battery assessment

Neuropsychological domain	Group D		Group I		Group M		Statistics	
	n	%	n	%	n	%	X^2	*p*
Attention / Executive function								
Alertness, TAP[a]	8/29	27.6%	11/38	28.9%	8/24	33.3%	0.22	0.893
Divided attention, TAP[a]	12/28	42.8%	21/37	56.7%	8/23	34.8%	2.98	0.225
Selective attention, TAP[a]	5/29	17.2%	9/38	23.7%	2/23	8.7%	2.21	0.331
Trail Making Part A, TMT[b]	10/29	34.5%	11/38	28.9%	4/24	16.6%	2.16	0.339
Trail Making Part B, TMT[b]	9/28	32.1%	11/38	28.9%	4/24	16.6%	1.75	0.415
Tower of London, TvL[c]	3/29	10.3%	6/38	15.8%	4/24	16.6%	0.54	0.759
Visuo-constructive functions								
Mental rotation, LPS[d]	6/28	21.4%	5/37	13.5%	3/24	12.5%	1.01	0.602
Visual-spatial function, 5PT[e]	6/30	20.0%	7/38	18.4%	2/24	8.3%	1.54	0.462
Verbal fluency								
Phonematic, RWT[f]	14/30	46.6%	14/38	36.8%	9/24	37.5%	0.77	0.679
Semantic, RWT[f]	8/30	26.6%	10/38	26.3%	7/24	29.2%	0.06	0.967
Learning / Memory								
Verbal learning/memory, VLMT[g]	17/30	56.6%	22/38	57.9%	12/24	50.0%	0.39	0.819
Visual digit span forward, BTT[h]	12/30	40.0%	15/38	39.5%	10/24	41.6%	0.03	0.984
Visual digit span backwards., BTT[h]	12/30	40.0%	17/36	47.2%	7/24	29.2%	1.51	0.469
Hand motor function								
Finger tapping right hand, FTT[i]	0/27	0%	0/38	0%	0/24	0%		
Finger tapping left hand, FTT[i]	1/27	3.7%	1/37	2.7%	0/24	0%	0.83	0.657
Finger tapping both hands, FTT[i]	3/27	11.1%	2/37	5.4%	0/24	0%	2.93	0.230

[a] Testbatterie zur Aufmerksamkeitsprüfung [22], [b] Trail Making Test [23], [c] Tower of London [24], [d] Leistungs-prüfsystem [25], [e] Five-Point-Test, [f] Regensburger Wortflüssigkeitstest [27], [g] Verbal Learning and Memory Test [28], [h] Block Tapping Test [29], [i] Finger Tapping Test [30]
